# Overexpression of endothelial S1pr2 promotes blood–brain barrier disruption via JNK/c-Jun/MMP-9 pathway after traumatic brain injury in both *in vivo* and *in vitro* models

**DOI:** 10.3389/fphar.2024.1448570

**Published:** 2024-11-29

**Authors:** Hongbo Cheng, Yijiao Men, Yaqing An, Jiegang Yu, Gengshen Zhang, Jiaming Li, Xiaoliang Wang, Guozhu Sun, Yang Wu

**Affiliations:** ^1^ Department of Neurosurgery, The Second Hospital of Hebei Medical University, Shijiazhuang, China; ^2^ Emergency Department, The Second Hospital of Hebei Medical University, Shijiazhuang, China

**Keywords:** traumatic brain injury, S1pr2, blood-brain barrier, endothelial cells, MMP-9

## Abstract

**Objectives:**

The disruption of blood-brain barrier (BBB) is associated with poor outcomes of TBI patients. Sphingosine-1-phosphate receptor 2 (S1pr2), a member of the G protein-coupled receptor family, is involved in endothelial activation and the regulation of vascular integrity. We hypothesized that the inhibition of S1pr2 may alleviate BBB disruption and explored potential underlying molecular mechanisms.

**Methods:**

Lesion volumes were assessed utilizing Nissl staining; neurological outcomes were evaluated through a battery of neurobehavioral assessments; phenotype-associated proteins were scrutinized via Western blot analysis; levels of reactive oxygen species (ROS), neuronal apoptosis, and S1pr2 expression were determined using immunofluorescence staining. The impact of S1pr2 inhibition after TBI and its underlying mechanism were elucidated using the selective S1pr2 inhibitor JTE-013, the JNK phosphorylation inhibitor SP600125, and cellular models. Chip-qPCR was employed to further elucidate the binding sites of the transcription factor c-Jun.

**Results:**

The expression of S1pr2 significantly increased following TBI in mice. Pharmacological inhibition of S1pr2 alleviated secondary injury with reduced lesion volume, ROS generation, cerebral oedema, neurological deficits, and neuronal apoptosis; BBB disruption was also mitigated, accompanied by reduced degradation of tight junction proteins and decreased induction of matrix metalloproteinases-9 (MMP-9) post-TBI. Mechanistically, TBI induces an increase in S1pr2 specifically in endothelial cells, leading to the promotion of MMP-9 transactivation by enhancing JNK/c-Jun signaling. This results in the degradation of tight junction proteins and increased BBB permeability. Through *in vitro* and *in vivo* Chip-qPCR experiments, we verified that AP-1a and AP-1b of MMP-9 promoter function as binding sites for phosphorylated c-Jun.

**Conclusion:**

Our findings identify a previously undisclosed role of S1pr2 in the pathophysiology of TBI. The S1pr2 inhibition presents a novel approach to alleviate BBB disruption after TBI through regulating the JNK/c-Jun/MMP-9 pathway.

## 1 Introduction

Traumatic brain injury (TBI) is a global public health issue with the incidence of TBI rising worldwide (Rigon et al., 2016). The blood-brain barrier (BBB) disruption is a hallmark of the pathogenesis of TBI (Readnower et al., 2010). The high mortality and disability rates associated with TBI are exacerbated by the disruption of the BBB, a critical mechanism that worsens secondary injuries post-TBI such as neuroinflammation, cerebral edema, and oxidative stress (Tchantchou and Zhang, 2013). Early administration of vasoprotective agents may potentially mitigate the risk and severity of these complications by enhancing the integrity of the damaged cerebral blood vessels, ultimately leading to improved outcomes in TBI (Sandsmark et al., 2019).

Matrix metalloproteinases (MMPs) are a family of zinc-dependent endopeptidases that play crucial roles in the remodeling of the extracellular matrix and are implicated in disrupting cerebrovascular integrity after TBI (Wei et al., 2011; Chen et al., 2018). During the acute phase of brain injury (minutes to hours post-event), the release of reactive oxygen species and pro-inflammatory cytokines within the brain parenchyma is known to trigger vascular permeability and the upregulation of MMP expression and activity (Lee et al., 2012). Within the MMP family, basal levels of MMP-9 are typically low but are swiftly induced by pro-inflammatory stimuli (Zhang H. et al., 2022). While the role of MMPs in neurovascular injury in TBI is well-established, there is currently a lack of therapies specifically targeting MMPs, primarily due to the intricate regulation of MMP activity and the non-specific nature of MMP inhibitors along with their myriad side effects. Hence, alternative targets are required to modulate MMP activity or BBB integrity.

The bioactive sphingolipid, sphingosine-1-phosphate (S1P), has emerged as a crucial mediator in the regulation of vascular integrity, drawing significant interest in the scientific community (Kitano et al., 2019). S1pr2, a receptor for S1P, has been documented to be present in vascular endothelial cells (Chen et al., 2022). The complex functions of S1pr2 have been clarified in neurological disease models, such as ischemic stroke and multiple sclerosis, demonstrating its role in regulating vascular permeability and facilitating inflammation (Chen et al., 2022; Seyedsadr et al., 2019). However, its precise role in TBI remains unclear. Building upon the aforementioned revelations, we postulate that S1pr2 may represent a novel therapeutic target for maintaining vascular equilibrium in the acute phase of TBI, thereby illuminating novel avenues for therapeutic interventions in neurovascular disorders post-TBI.

Herein, we sought to evaluate the role of S1pr2 in maintaining cerebrovascular integrity following TBI. Using a mouse TBI model, we elucidated the critical role of S1pr2 in the disruption of BBB after TBI. Our results revealed that S1pr2 was induced in cerebrovascular endothelial cells early after TBI. JTE-013, a specific inhibitor of S1pr2, has been demonstrated to attenuate BBB damage and improve outcomes after TBI. Notably, S1pr2-mediated BBB disruption was associated with increased MMP-9 expression in the endothelial cells. Furthermore, we demonstrated that S1pr2 promoted MMP-9 expression through regulating the JNK/c-Jun pathway. Collectively, our data identified S1pr2 as a promising therapeutic target for preserving cerebrovascular integrity in the context of TBI.

## 2 Materials and methods

### 2.1 Animals and ethical considerations

Male wild-type (WT) C57BL/6 mice (20–25 g, 8–12 weeks old) were purchased from the Animal Center of Hebei Medical University. Animals were housed in a specific pathogen-free environment animal room with a 12-h light/dark cycle, a constant temperature of 23°C and humidity of 60%. The animals had *ad libitum* access to water and food from at least 1 week before the experiments. All experimental procedures were approved by the Ethics Committee of Hebei Medical University. Animal experiments were performed in accordance with the guidelines of the National Institutes of Health Guide for the Care and Use of Laboratory Animals.

### 2.2 TBI surgery, drug administration and experimental design

The TBI model was established using a controlled cortical impact (CCI) device (68,099 precision strike; RWD). Briefly, the mice were anaesthetized using 2% pentobarbital sodium, and the head was fixed on a stereotactic device. Craniotomy was performed over the right parietal bone window (1.5 mm towards the midline and 1.5 mm behind the bregma) using a 2-mm-diameter dental drill. A flat metal tip was used to strike the cortex at a speed of 3 m/s and depth of 1 mm; the contact time was 0.8 s. Cyanoacrylate tissue glue was used to close the scalp. The sham control group underwent craniotomy but did not receive CCI injury. To investigate the impact of S1pr2 antagonists following TBI, we administered intraperitoneal JTE-013 (10 mg/kg) dissolved in dimethyl sulfoxide (DMSO) to mice 30 min post-TBI induction (Seyedsadr et al., 2019). Mice were blindly randomized into different groups: (a) sham group, (b) TBI group, (c) TBI + JTE-013 group.

### 2.3 RNA sequencing and processing

RNA was extracted, sequenced, and analyzed by a custom service provided by Gene Denovo Biotechnology Co. (Guangzhou, China). High-quality sequencing data were mapped to the mouse reference genome using Bowtie2. The expression levels and variations of each gene were then normalized to fragments per kilobase of transcript per million mapped reads (FPKM) using RNA-seq by Expectation Maximization (RSEM). We identified differentially expressed genes (DEGs) between samples and performed principal component analysis (PCA) using the OmicShare tools at www.omicshare.com/tools.

### 2.4 RNA extraction and RT-PCR

RNA was extracted using TRIzol^®^ reagent based on the manufacturer’s guidelines (Vannini et al., 2017). The concentration and purity of RNA were evaluated by Nanodrop spectroscopy (Thermo Fisher Scientific, MA, United States). cDNA was synthesized from 1 μg RNA using the PrimeScript RT kit. Quantitative PCR system (CFX ConnectTM Real-Time System, Bio-Rad, CA, United States) and SYBR^®^ Green PCR Master Mix (Applied Biosystems, United States) were used for qRT-PCR. The cycle program was initially maintained at 95°C for 5 min, then denatured at 95°C for 40 cycles, annealed at 62°C for 30 s, and extended at 72°C for 30 s. The mRNA expression level of the target gene was normalized to the mRNA expression level of the housekeeping gene β-actin, and analyzed by the 2^−ΔΔCT^ method. The oligonucleotide PCR primer pairs purchased from Sangon Biotech (Shanghai, China) are listed in Supplementary Table S1.

### 2.5 Western blotting

Western blotting was performed as described previously (Poulet et al., 2020). Samples of the mouse tissue and endothelial cells were sonicated and homogenized by an ultrasonic homogenizer in the lysis buffer comprising 1% protease and phosphatase inhibitor (Roche, Mannheim, Germany). The extracted proteins were separated by SDS-PAGE and transferred onto PVDF membranes. After blocking with 5% non-fat milk, the membranes underwent incubation with primary antibodies targeting Erk: anti-Erk and anti-phosphorylated Erk (1:1,000; #4370/#4695, CST), JNK: anti-JNK and anti-phosphorylated JNK (1:1,000; #9251/#9252, CST), p38: anti-p38 and anti-phosphorylated p38 (1:1,000; #9212/#9211, CST), c-Jun: Anti-c-Jun and anti-phosphorylated-c-Jun (1:1,000; #9165/#2361), anti-MMP-9 (1:1,000; ab76003; Abcam), anti-S1pr2 (1:500, 21180-1-AP, Proteintech), anti-Occludin (1:1,000, ab216327, Abcam); anti-BAX (1:1,000, 50599-2-Ig, Proteintech); anti-Bcl-2 (1:1,000, 68103-1-Ig, Proteintech); anti-ZO-1 (1:500, ab96587, Abcam); anti-Occludin (1:1,000, 27260-1-AP, Proteintech); anti-β-actin (1:3,000, AC026, ABclonal). Membranes were then incubated with horseradish peroxidase (HRP)-conjugated secondary antibodies. Images were captured using a Bio-Rad Imaging System, and the protein bands were analyzed using ImageJ.

### 2.6 Immunofluorescence for S1pr2 detection and TUNEL staining

Mice were anesthetized using 2% pentobarbital and subsequently perfused with cold PBS followed by a 4% PFA in PBS solution. The brains were then extracted, post-fixed in 4% PFA for 24 h, and transferred to a 30% sucrose solution. Frozen brain samples were sliced to a thickness of 30 μm. The brain slices underwent three washes with Tris-buffered saline (TBS) and were then blocked using a TBS blocking solution (composed of 1% bovine serum albumin, 0.2% skim milk, and 0.3% Triton X-100 in TBS) for 1 h. Following this, the slices were incubated overnight on a shaker at 4°C with the primary antibodies, rabbit anti-S1P2R antibody (1:100, Proteintech Group Inc.) and rat anti-CD31 antibody (1:100, BD Biosciences, Pharmingen), in the TBS blocking solution. Subsequently, the sections were washed three times with TBS and then incubated with corresponding secondary antibodies. DAPI staining was performed for 7 min on the brain slices, which were then mounted onto slides. Imaging of all sections was carried out using an A1 Si confocal microscope (Nikon), and the analysis was conducted using ImageJ software.

To assess apoptosis levels, brain sections underwent TUNEL staining with a TUNEL Apoptosis Assay kit (Zhang W. et al., 2021). Tissue samples were fixed in 4% paraformaldehyde at 4°C for 24 h, paraffin-embedded, dewaxed, and subjected to the TUNEL assay following the manufacturer’s protocol. The slices were then incubated with Rabbit anti-NeuN antibody (1:200, Sigma, ABN90P) conjugated with Alexa Fluor 488 (1:200, A-11055, Invitrogen). DAPI Fluoromount-G™ anti-fluorescence quenching sealant was utilized for slide sealing. The analysis involved six brain samples from each experimental group, with a minimum of four microscopic fields captured around the injured area of each brain sample. TUNEL and Neun-positive cells were quantified in a double-blinded fashion. The mean ratio of TUNEL^+^Neun^+^ to Neun^+^ cells from these regions was calculated to determine the extent of apoptosis.

### 2.7 Measurement of cerebral water content and quantification of lesion volume

The measurement of brain water content was performed using whole brain tissues (Wu et al., 2023). The wet-dry ratio was calculated as a percentage using the following equation: brain water content (%) = (wet weight − dry weight)/wet weight × 100%.

Lesion volumes were quantified as described previously (Lee et al., 2014). Mice were sacrificed 72 h after TBI, and brain sections were prepared. Thereafter, Nissl bodies were stained using cresyl violet, and ImageJ was used to quantify the lesion volume in each brain section.

### 2.8 Determination of reactive oxygen species (ROS) levels

Dihydroethidium (DHE) staining was used to evaluate intracellular ROS levels in post-TBI brains (Wu et al., 2020). Briefly, each brain was cut into 30-μm-thick sections, which were then stained using DHE (Yesen, 50102ES02) for 30 min and imaged using a laser scanning confocal microscope (A1 Si, Nikon).

### 2.9 Modified neurological severity score (mNSS) test

The mNSS score was a composite of the motor, sensory, balance and reflex tests, as described in a previous study (Zhang Q. et al., 2022). This test was performed at 3 days post-TBI and assessed by experimenters who were blinded to the conditions.

### 2.10 Rotarod test

An automated rotarod (San Diego Instruments, San Diego, CA, United States) was used to assess the limb motor coordination and balance of mice after trauma (Wang C. et al., 2022). Mice in each group received 3 days of behavioral training before TBI induction. Briefly, mice were placed on an accelerating rotating rod, which accelerated from 5 to 40 rpm/min within 5 min. The latency to fall for each mouse was recorded. This test was conducted before TBI and at 1, 3, 7, and 14 days after TBI and was scored by blinded experimenters.

### 2.11 Cell culture and drug administration

hCMEC/D3 cells, as a model of human brain endothelium, were cultured in Endothelial Cell Basal Medium-2 (EBM-2) (Lonza, Allendale, NJ, United States) supplemented with the EGMTM- 2 BulletKit™ (Lonza, Allendale, NJ, United States) (Poulet et al., 2020). Cells were cultured in an incubator with 5% CO2 and 95% air, and maintained at 37°C. To mimic *in vitro* metabolic and inflammatory stress of TBI injury, LPS, oxygen-glucose deprivation (OGD) and TNF-a activation studies were conducted. To identify the effects of JTE013 and SP600125 on TNF-α-induced hCMEC/D3 cells and the possible mechanisms, the cells were exposed to TNF-α (5 ng/mL), S1pr2 antagonist JTE013 (1 μM) and JNK inhibitor SP600125 (40 μM).

### 2.12 Cell transfection

Small interfering RNA (siRNA) against c-Jun and scramble siRNA were purchased from Zhonghong Boyuan (Shenzhen) Biotechnology Co., Ltd. The sequences of c-Jun siRNA are listed in Supplementary Table S2. siRNAs were transfected into cells using Lipofectamine 2000 (Invitrogen) according to the manufacturer’s instructions. pcDNA3.1-S1pr2 overexpression vector and pcDNA3.1 empty vector were constructed and synthesized by Zhonghong Boyuan (Shenzhen) Biotechnology Co., Ltd. The overexpression of S1pr2 was achieved with the pcDNA3.1-S1pr2 vector (S1pr2), and the pcDNA3.1 empty vector was used as a negative control. Plasmid DNA was complexed with Lipo 2000 according to the provided instructions and subsequently introduced into the cell culture medium. The protein expression of the target gene was then assessed 48 h later.

### 2.13 Isolation of brain CD31^+^ endothelial cells

Adult mouse brain endothelial cells were isolated as previously described using enzymatic dissociation and anti-CD31–conjugated magnetic beads (Hoffmann et al., 2015). Mice were transcardially perfused with PBS, and brains were dissociated using the Papain Neural Tissue Dissociation Kit (Miltenyi Biotec). Myelin was removed and cells were labeled with anti-CD31 microbeads™ (Miltenyi Biotec). We conducted Magnetic Activated Cell Sorting (MACS) isolation in accordance with the manufacturer’s guidelines. Cells were frozen at −80°C until processing for western blot.

### 2.14 Chromatin immunoprecipitation (ChIP) assays

Isolated mouse brain endothelial cells were utilized for each Chromatin Immunoprecipitation (ChIP) analysis (Winkler et al., 2021; Haynes et al., 2018). The cells underwent cross-linking with 1% formaldehyde at 37°C for a duration of 10 min. Following PBS washing, the cells were reconstituted in 300 μL of lysis buffer. Subsequently, DNA fragmentation was achieved through sonication. The obtained supernatants were subjected to a 2-h incubation with anti-phosphorylated c-Jun (Santa Cruz, sc-16312) or an isotype control IgG in the presence of Protein A/G Magnetic beads (Thermo Fisher). The immunoprecipitated DNA was eluted from the beads using 1% SDS and a 1.1 M NaHCO_3_ solution at 65°C for 6 h. Purification of the DNA was then performed utilizing a PCR Purification Kit (QIAGEN, United States). Brain tissues from the TBI cortex or sham cortex were finely minced using a sterile razor blade in liquid nitrogen. Proteins were cross-linked in a freshly prepared formaldehyde solution at ambient temperature for a duration of 10 min. Prior to usage, a protease inhibitor cocktail comprising one tablet per 10 mL (Roche, Basel, Switzerland) was introduced into the immunoprecipitation lysis buffer. The following steps are similar to those of the previous cell samples. The primer sequences are listed in Supplementary Table S3.

### 2.15 Statistical analysis

Statistical analyses were conducted utilizing GraphPad Prism 8.0 (La Jolla, CA). The data were depicted as the mean ± standard deviation (SD). Unpaired two-tailed Student’s t-test was employed to compare two independent groups, while one-way analysis of variance was utilized for comparing multiple groups. Two-way ANOVA was conducted for the analysis of experimental data involving two factors, followed by Bonferroni *post hoc* testing. A significance level of *p* < 0.05 was considered statistically significant.

## 3 Result

### 3.1 RNA-seq uncovered a notable rise in the expression levels of the S1pr2 after TBI in mice

**FIGURE 1:**
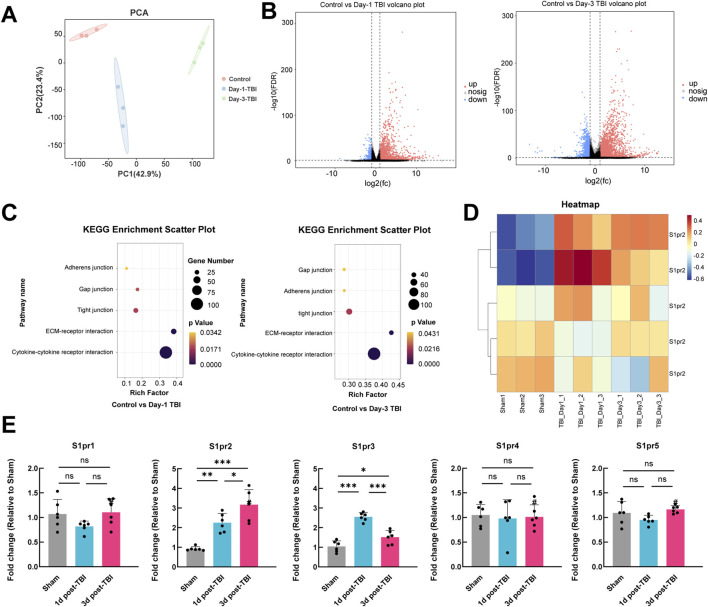
RNA sequencing uncovered a notable rise in the expression levels of the S1pr2 after TBI in mice. **(A)** PCA plot illustrating the separation of samples along the first two principal components. **(B)** Volcano plot comparing control vs*.* 1 day after TBI samples, and comparing control vs*.* 3 days after TBI samples. **(C)** KEGG enrichment analysis demonstrating the changes in gene expression in control vs*.* 1 day after TBI samples, and control *versus* 3 days after TBI samples. **(D)** Heatmap depicting the expression levels of S1pr2 family genes across different groups. **(E)** qPCR analysis of S1pr gene family members at 1 and 3 days after TBI (n = 6 per group). Data are presented as mean ± SD. *p < 0.05; **p < 0.01; ***p < 0.001, by one-way ANOVA **(E)**.

We used RNA-seq to analyze mRNA expression following TBI in both TBI-injured and control brain tissues, with the goal of identifying potentially influential molecules. PCA analysis was conducted on all samples, and the results are depicted in Figure 1A. The Transcription characteristics of the three groups were significantly separated, which suggests that TBI results in complex molecular changes in the pathological process after TBI. The volcano plot displayed the overall up- and down-regulated molecules in both groups at 1-day and 3-day post-TBI. The results showed that there was a higher number of differentially expressed genes at 3 days post-TBI compared to 1 day post-TBI (Figure 1B). Interestingly, Kyoto Encyclopedia of Genes and Genomes (KEGG) pathways showed adherens junction, gap junction, tight junction, ECM-receptor interaction, and cytokine-cytokine receptor interaction were significant pathways both at 1-day and 3-day post-TBI, highlighting the crucial role of the cell junction associated pathway in the pathological process after TBI (Figure 1C). The heat map illustrated the changes of the S1pr2 family after TBI, revealing a significant increase in S1pr2 and S1pr3 levels following TBI (Figure 1D). Subsequently, the S1pr family was further analyzed by the qPCR. The results showed that on day 1 post-TBI, the TBI group displayed a substantial upregulation of S1pr2 and S1pr3 at the transcriptional level compared to the sham cohort; on day 3 post-TBI, there was a notable increase in S1pr2 levels, while S1pr3 expression exhibited a pronounced decline compared to day 1 post-TBI. The levels of S1pr1, S1pr4, and S1pr5 showed no changes following TBI (Figure 1E). These findings suggest intercellular tight junctions were involved in the pathological progression of TBI and S1pr2 is persistently elevated during the acute phase of TBI.

### 3.2 The expression of S1pr2 was upregulated in endothelial cells after TBI

**FIGURE 2:**
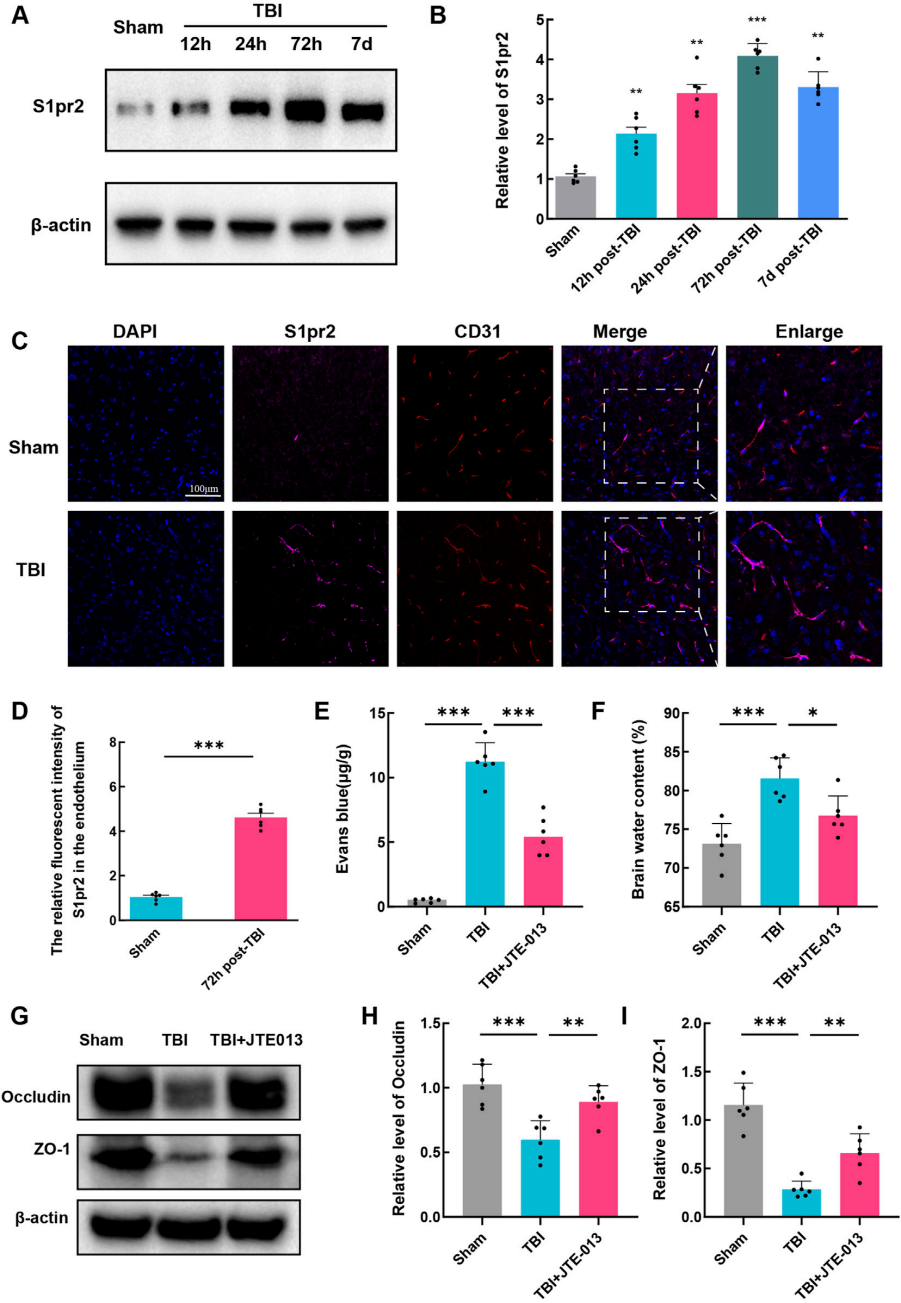
The expression of S1pr2 was upregulated in endothelial cells after TBI. **(A, B)** Western blot analysis of S1pr2 expression at different time points after TBI (n = 6 per group). **(C)** Immunofluorescence staining of S1pr2 in endothelial cells in sham and TBI groups. Scale bar, 100 μm. Merged images show the colocalization of S1pr2 and CD31 at the contusion area, along with enlarged images (n = 6 per group). **(D)** Quantification analysis of the relative fluorescence intensity in endothelial cell (6 fields of view, n = 6 per group). **(E)** Quantification of Evan Blue in each group (n = 6 per group). **(D)** Quantification of brain water content at 3 days after TBI (n = 6 per group). **(G, I)** Western blot detecting the expression of Occludin and ZO-1 in each group, and the related statistical analysis. Data are presented as mean ± SD. *P < 0.05, **P < 0.01, ***P < 0.001, by one-way ANOVA **(B, E, F, H, I)** and unpaired two-tailed *t*-test **(D)**.

To further explore the potential involvement of S1pr2 in TBI, we examined changes in S1pr2 protein expression following TBI. We analyzed the variations in S1pr2 levels in the perilesional brain tissue at different time points after TBI in mice. Our investigation revealed a steady increase in S1pr2 expression at 12 h, reaching its peak at 72 h, and gradually decreasing until 7 days (Figures 2A, B). Due to the significant elevation of S1pr2 levels at 72 h post-TBI, we selected this time point as the post-TBI reference for subsequent experiments.

We also revealed the significant increase of S1pr2 in endothelial cells following TBI by immunofluorescence staining (Figures 2C, D). Subsequent investigations were thus focused on elucidating the role of S1pr2 in TBI-induced endothelial alterations. Due to the involvement of vascular endothelial cells in BBB impairment, a pivotal process in TBI-induced secondary brain injury, our subsequent investigations aimed to detect brain edema and Evans blue extravasation to assess blood-brain barrier integrity. To validate the role of S1pr2 on BBB impairment, mice were treated with JTE013, a specific inhibitor of S1pr2, after TBI. Notably, TBI promoted Evans blue extravasation in brain tissues compared to the sham group, whereas the administration of JTE013 significantly mitigated this elevation relative to the TBI group, suggestive of JET013’s capacity to safeguard BBB integrity (Figure 2E). Consistent with this observation, our findings revealed that JTE-013 also notably attenuated brain edema post-TBI (Figure 2F). Additionally, in our Western blot analyses of BBB-related proteins, we observed a reduction in the expression of tight junction proteins (Occludin and ZO-1) in the TBI group compared to the sham group, which was significantly reversed by JET013 treatment (Figures 2G–I).

### 3.3 Inhibition of S1pr2 mitigated oxidative stress, neural apoptosis, lesion volume, and neurological functional deficits following TBI

**FIGURE 3:**
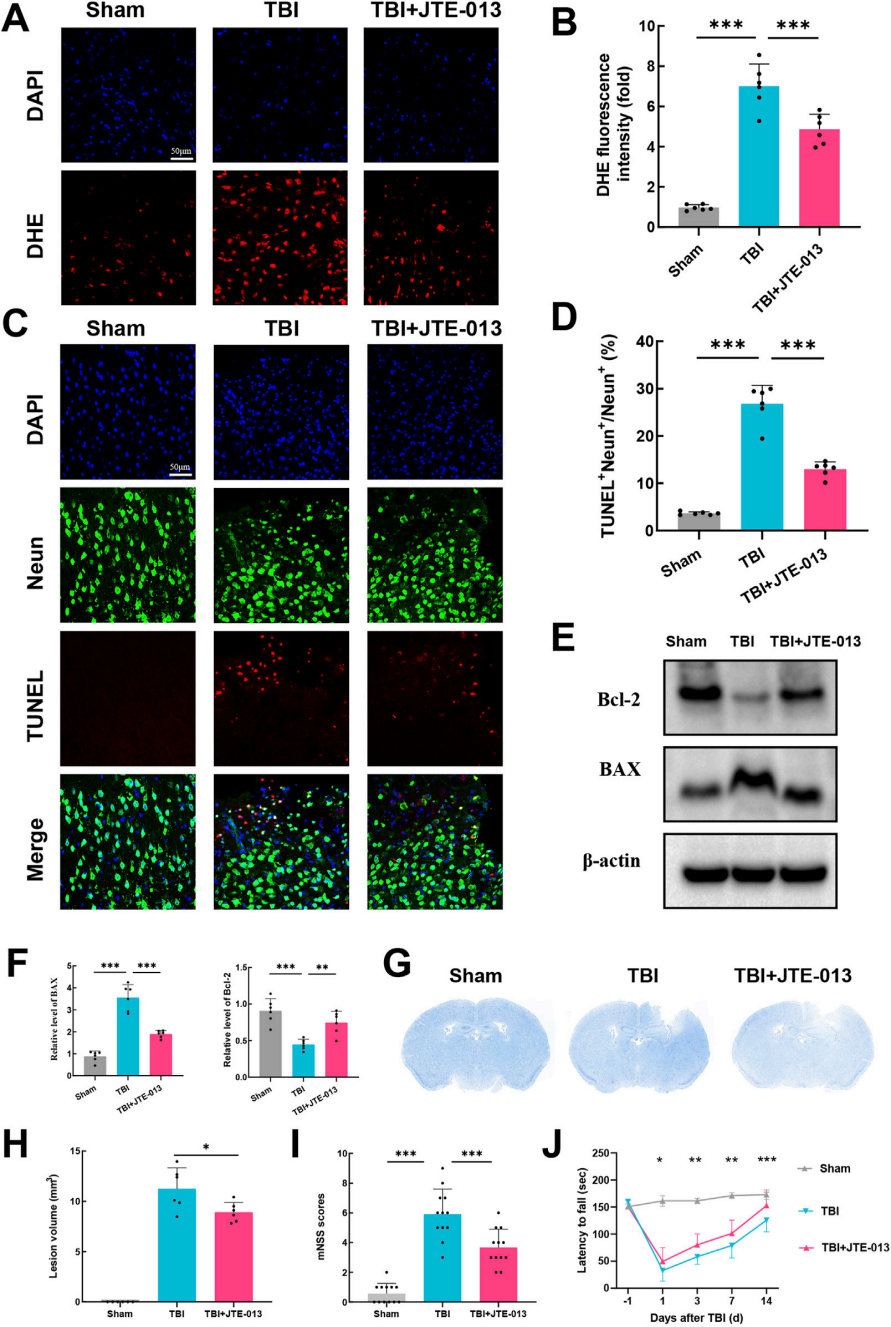
Inhibition of S1pr2 mitigated oxidative stress, neuronal apoptosis, lesion volume, and neurological functional deficits following TBI. **(A, B)** DHE staining and statistical analysis of fluorescence intensity at 3 days after TBI. Scale bar, 50 μm (n = 6 per group). **(C, D)** Representative images of TUNEL staining and statistical analysis at 3 days after TBI (n = 6 per group). Scale bar, 50 μm. **(E, F)** Western blot analysis of BAX and Bcl-2 (n = 6 per group). **(G, H)** Representative images of Nissl staining and quantification of lesion volumes at 3 d post-TBI (n = 6 per group). **(I)** Neurological deficit was assessed by mNSS on day 3 after TBI (n = 12 per group). **(J)** Motor function was evaluated using rotarod test before TBI and at 1, 3, 7, and 14 days after TBI (n = 12 per group). Data are presented as mean ± SD. *P < 0.05, **P < 0.01, ***P < 0.001, by one-way ANOVA (B, D, F, H and I) and two-way ANOVA **(J)**.

First, we assessed reactive oxygen species (ROS) levels using dihydroethidium (DHE) staining, where increased fluorescence intensity signifies elevated ROS levels. DHE fluorescence intensity was significantly heightened post-TBI, whereas in the TBI + JTE-013 group, it was notably lower compared to the TBI groups (Figures 3A, B). It is widely recognized that oxidative stress plays a pivotal role in neural apoptosis and the worsening of conditions after TBI. TUNEL staining in TBI group mice revealed a substantial increase in neural apoptosis post-TBI, while treatment with JTE-013 markedly decreased neural apoptosis following TBI (Figures 3C, D). Western blot analysis showed a significant elevation in BAX levels and a reduction in Bcl-2 levels post-TBI, which were reversed by JTE-013 treatment (Figures 3E, F). These findings indicate that JTE-013 treatment diminishes oxidative stress and neural apoptosis following TBI.

Furthermore, administration of JTE-013 resulted in a significant decrease in lesion volume following TBI by Nissl staining using cresyl violet (Figures 3G, H). The modified neurological severity score (mNSS) was used for neurological function assessment after TBI in mice. Using mNSS score, TBI group exhibited elevated neurological functional deficit scores on day 3, whereas those treated with JTE-013 demonstrated lower deficit scores relative to the TBI group (Figure 3I). Notably, in the grid-walking evaluation, the TBI group displayed a pronounced increase in foot faults compared to the sham group at 1, 3, 7, and 14 days post-TBI, whereas the TBI + JTE-013 group exhibited a substantial reduction in foot faults compared to the TBI group (Figure 3J). These findings suggest that JTE-013 treatment confers neuroprotective benefits in TBI models.

### 3.4 MMP-9 contributes to the deleterious effects of S1pr2 on TBI

**FIGURE 4:**
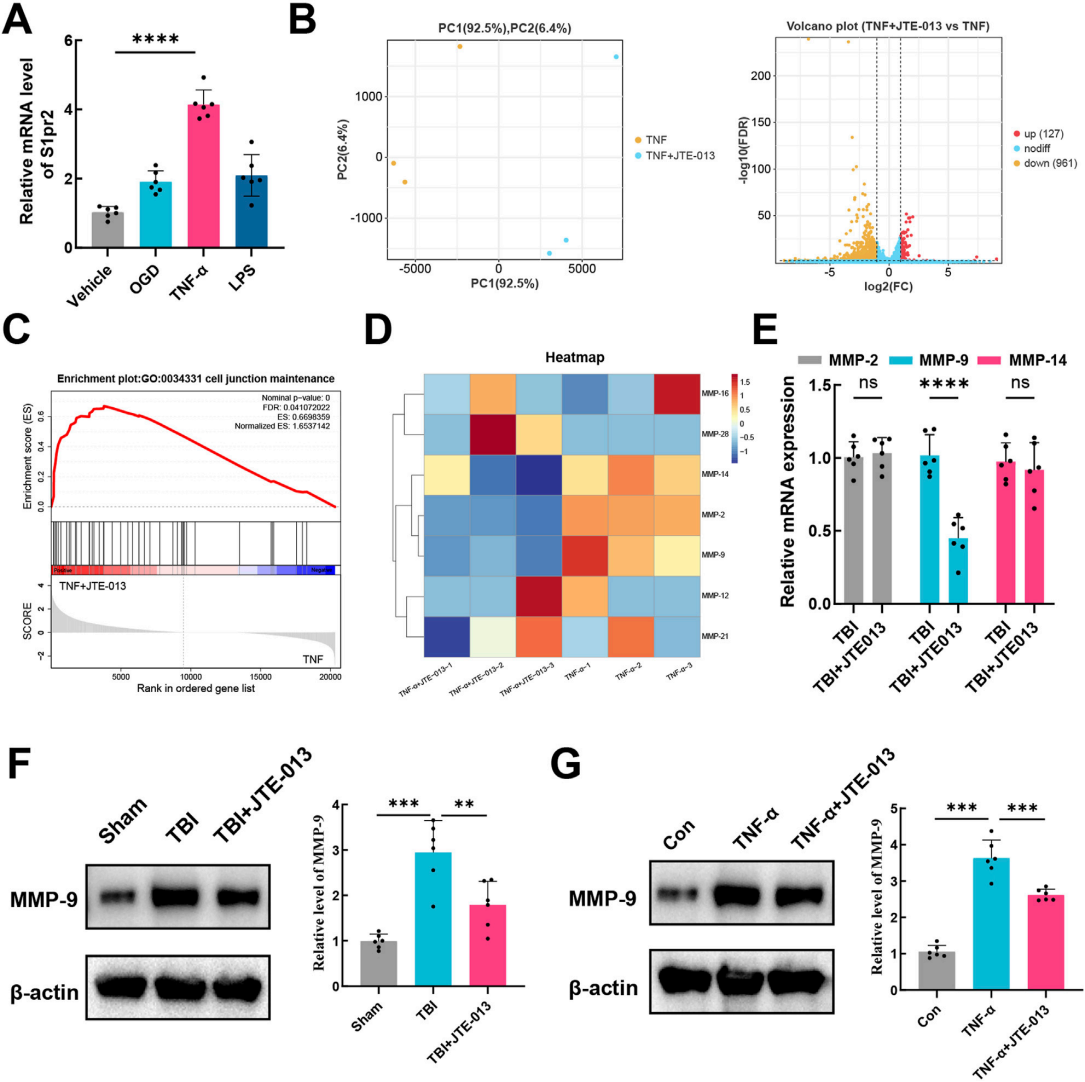
MMP-9 confers the deleterious effects of S1pr2 on TBI. **(A)** qPCR analysis of mRNA level of S1pr2 under different experimental conditions *in vitro* (n = 6 per group). **(B)** PCA and the volcano plot illustrated the global up- and down-regulated genes in the TNF-α group and the TNF-α + JTE-013 group (n = 3 per group). **(C)** Gene Set Enrichment Analysis (GSEA) highlighted the crucial role of S1pr2 in regulating cell junction. **(D)** The heat map displayed alterations in MMP family members (n = 3 per group). **(E)** qPCR analysis of MMP-2, MMP-9, and MMP-14 after TBI with or without JTE-013 treatment (n = 6 per group). **(F)** Western blot analysis of MMP-9 *in vivo* (n = 6 per group). **(G)** Western blot analysis of MMP-9 *in vitro* (n = 6 per group). hCMEC/D3 endothelial cells were treated with 5 ng/mL TNF-a in the presence or absence of the S1pr2 antagonist JTE013 (1 μM) for 24 h. *In vitro* experiments were conducted in the presence of serum, which contains S1P. Data are presented as mean ± SD. *p < 0.05, **p < 0.01, ***p < 0.001, by one-way ANOVA **(A, F, G)** and unpaired two-tailed *t*-test **(E)**.

The hCMEC/D3 cerebrovascular endothelial cell line is commonly utilized as a surrogate for establishing a human blood-brain barrier *in vitro*. These cells exhibit endothelial protein markers, tight junction proteins, efflux transporters, and other characteristic proteins present in human brain endothelial cells (Lecuyer et al., 2012). Experimental conditions mimicking a TBI model were applied to these endothelial cells, including OGD, TNF-α and LPS. It was observed that neither OGD nor LPS elicited an activating effect on S1pr2 in hCMEC/D3 cells. Conversely, cells treated with TNF-α exhibited a significantly elevated expression of S1pr2. TNF-α stimulation led to an approximately four-fold increase in S1pr2 expression compared to untreated samples (Figure 4A). To elucidate the potential mechanism by which S1pr2 activation may contribute to the dysfunction of brain endothelial cells, RNA-seq was employed to profile mRNA expression following TNF-α and JTE-013 treatment. Principal component analysis (PCA) results suggest a distinct separation pattern between the TNF-α group and the TNF-α + JTE-013 group. The volcano plot illustrated the global up- and downregulated molecules in the two experimental groups (Figure 4B). Remarkably, Gene Set Enrichment Analysis (GSEA) highlighted the crucial role of S1pr2 inhibition in preserving cell junction (Figure 4C). Given the pivotal involvement of the MMP family in augmenting cerebrovascular permeability post-TBI (Muradashvili et al., 2015), the heat map displayed alterations in MMP family members, with a noticeable decrease in MMP-9, MMP-2, and MMP-14 levels after JTE-013 treatment under TNF-α stimulations (Figure 4D). Subsequently, these members of the MMP family were selected for further *in vivo* investigation. qPCR results demonstrated a significant reduction in MMP-9 transcription in the TBI + JTE-013 group compared to the TBI group, while no substantial changes were observed in MMP-2 and MMP-14 levels between the two groups, indicating that inhibition of S1pr2 effectively suppressed TBI-induced MMP-9 activation (Figure 4E). Additionally, protein analysis revealed that TBI led to an upregulation of MMP-9 expression, which was mitigated by JTE-013 in the mouse TBI model (Figure 4F). Consistently, *in vitro* experiments also confirmed that S1pr2 inhibition significantly suppressed TNF-α-induced MMP-9 activation (Figure 4G). These data indicated that S1pr2-induced BBB disruption was associated with increased MMP-9 expression in the endothelial cells.

### 3.5 The activation of S1pr2 exacerbates BBB damage through the JNK pathway

**FIGURE 5:**
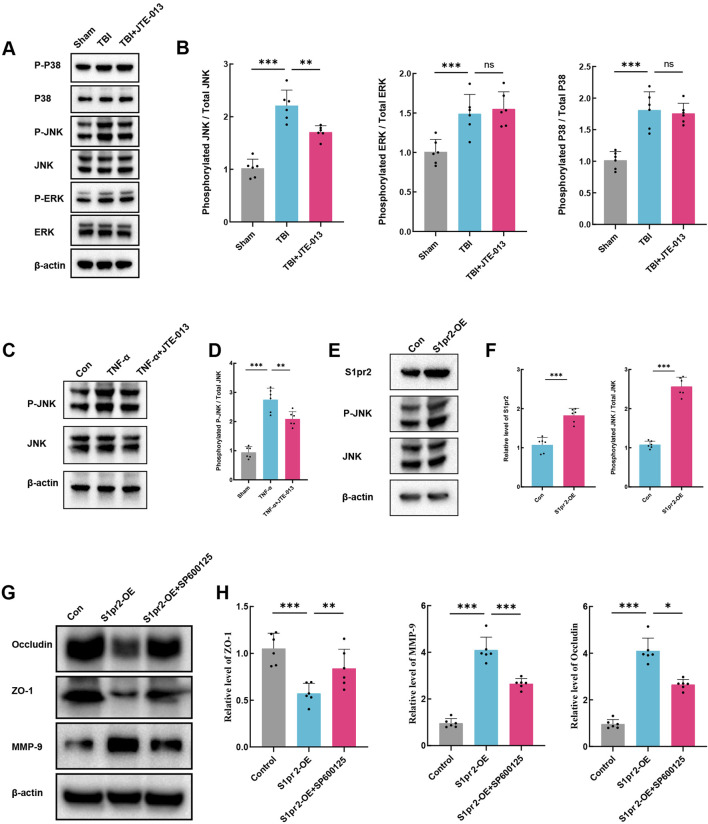
The activation of S1pr2 exacerbates BBB damage through the JNK pathway. **(A, B)** Western blot analysis of P-P38, P38, P-JNK, JNK, P-ERK, and ERK from each group at 3 days after TBI with or without JTE-013 treatment (n = 6 per group). **(C, D)** Western blot analysis of P-JNK and JNK *in vitro*. hCMEC/D3 endothelial cells were treated with 5 ng/mL TNF-α in the presence or absence of the JNK inhibitor SP600125 (40 μM) for 24 h (n = 6 per group). **(E, F)** Western blot analysis of S1pr2, P-JNK and JNK *in vitro* in each group (n = 6 per group). **(G, H)** Western blot analysis of Occludin, ZO-1 and MMP-9 *in vitro* in each group (n = 6 per group). *In vitro* experiments were conducted in the presence of serum, which contains S1P. Data are presented as mean ± SD. *P < 0.05, **P < 0.01, ***P < 0.001, by one-way ANOVA **(B, D, H)**.

Previous studies have indicated that the process of vascular destruction following TBI is associated with the upregulation of the MAPK pathway (Chai and Liu, 2007; Lan et al., 2019). The JNK/ERK/P38 signaling cascade serves as the crucial intermediary of the MAPK pathway. First, an analysis of the levels of the JNK/ERK/P38 proteins and their phosphorylation levels was conducted. The results demonstrated a significant elevation in the relative phosphorylation levels of JNK/ERK/P38 following TBI. Following treatment with JTE-013, the phosphorylation level of JNK notably decreased, while there was no significant alteration in the phosphorylation levels of ERK and P38 (Figures 5A, B). This suggests that the inhibition of S1pr2 may suppress the JNK phosphorylation pathway after TBI. Similarly, consistent with results observed *in vivo* experiments, JTE-013 treatment also significantly alleviated the phosphorylation level of JNK after TNF-α stimulation (Figures 5C, D). These findings substantiated the pivotal effect of S1pr2 on JNK phosphorylation *in vitro* model. To further investigate the effect of S1pr2 on JNK phosphorylation, cells were transfected with either an S1pr2 overexpression (S1pr2-OE) vector or an empty vector as a control. As anticipated, we observed a marked increase in JNK phosphorylation in the S1pr2-OE group compared to the control group (Figures 5E, F). This suggests that S1pr2 activation could activate the JNK pathway. To validate the role of S1pr2 activation in regulating MMP-9 expression via the JNK pathway, we introduced SP600125, a JNK phosphorylation inhibitor, into the *in vitro* investigations. The results showed that overexpression of S1pr2 led to a significant decrease in the expression of ZO-1 and Occludin, while increasing the expression of MMP-9. However, the SP600125 treatment exhibited a significant reversal of these trends (Figures 5G, H). These results suggest that the activation of S1pr2 may regulate BBB damage through the JNK phosphorylation pathway.

### 3.6 S1pr2 promotes MMP-9 expression via the JNK/c-jun pathway

**FIGURE 6:**
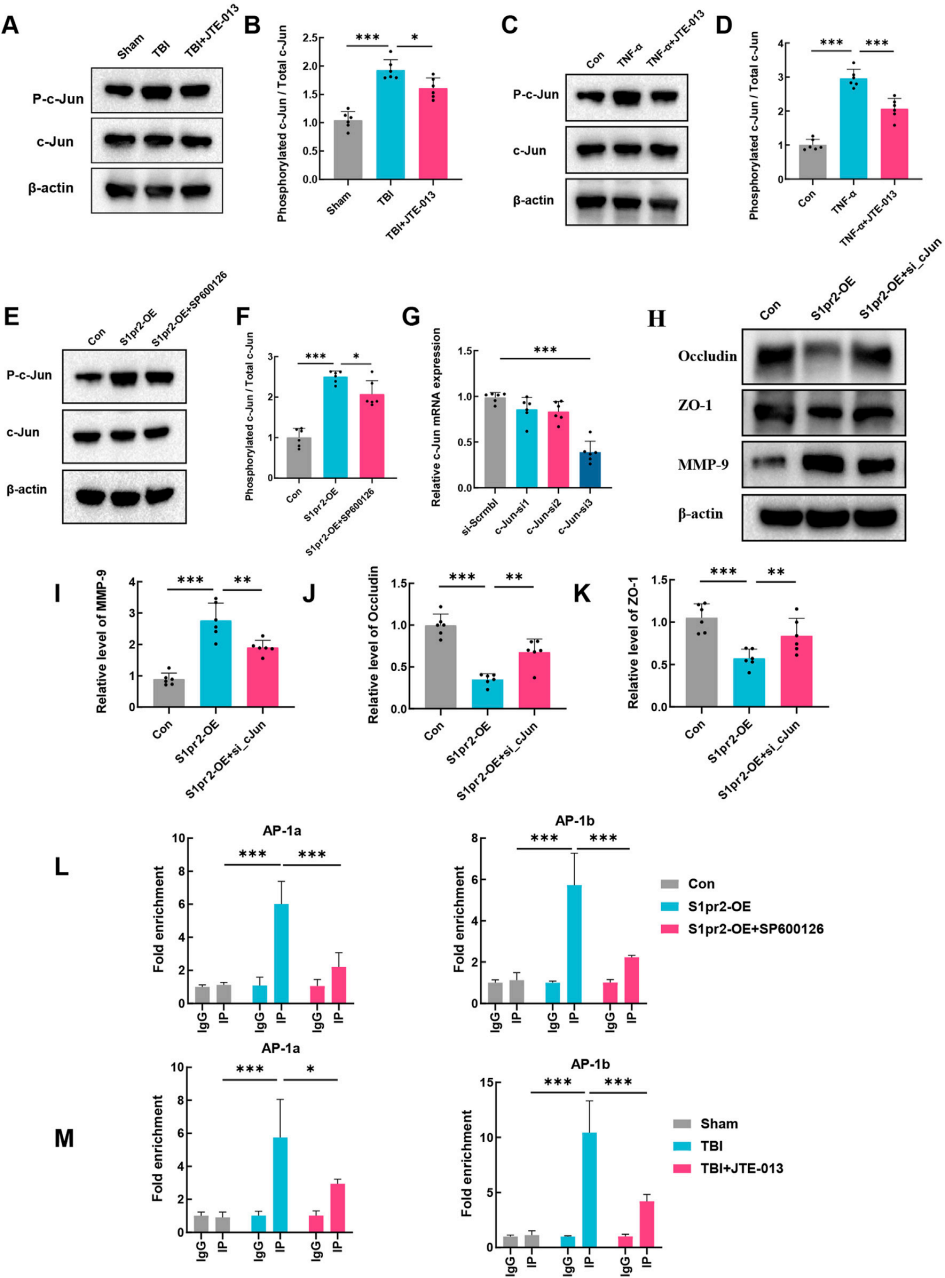
S1pr2 promotes MMP-9 expression via the JNK/c-Jun pathway. **(A, B)** Western blot analysis of P-c-Jun and c-Jun in each group at 3 days after TBI (n = 6 per group). **(C, D)** Western blot analysis of P-c-Jun and c-Jun in hCMEC/D3 endothelial cells treated with TNF-α in the presence or absence of the JTE013 (n = 6 per group). **(E, F)** Western blot analysis of P-c-Jun and c-Jun *in vitro* experimental conditions (n = 6 per group). **(G)** qPCR analysis of c-Jun following siRNA-mediated knockdown of c-jun in hCMEC/D3 endothelial cells. **(H–K)** Western blot analysis of Occludin, ZO-1 and MMP-9 *in vitro* experimental conditions. **(L)** Assessment of p-c-Jun binding to AP-1 sites within the MMP-9 promoter in primary mouse brain endothelial cells using the ChIP assay. **(M)** Assessment of p-c-Jun binding to AP-1 sites within the MMP-9 promoter in the brain of mice using the ChIP assay. Data are presented as mean ± SD. *p < 0.05, **p < 0.01, ***p < 0.001, by one-way ANOVA **(B, D, F, G, I–K)** and two-way ANOVA **(L, M)**.

**FIGURE 7:**
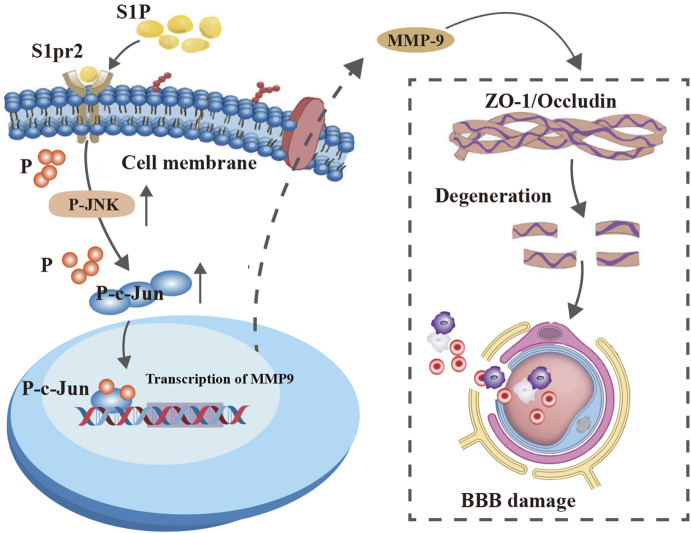
A graphic conclusion. S1pr2 activation in endothelial cells after TBI promoted MMP-9 expression by enhancing the JNK/c-Jun signaling pathway, thereby aggravating BBB disruption.

The c-Jun protein, a member of the activation protein 1 (AP-1) family, is known to be modulated by JNK phosphorylation (Hu et al., 2017). In addition, AP-1 regulates the transactivation of murine MMP-9 (Cho et al., 2007; Hwang et al., 2010). Thus, the possibility was explored that S1pr2 triggers MMP-9 expression during TBI by activating the JNK/c-Jun signaling pathway. Our findings revealed a marked increase in the phosphorylation ratio of c-Jun in endothelial cells following TBI compared to the sham group. Conversely, the TBI + JTE-013 group exhibited a significant decrease in the phosphorylation ratio of c-Jun (Figures 6A, B). Likewise, our *in vitro* experiments yielded parallel outcomes (Figures 6C, D). Meanwhile, we employed the JNK phosphorylation inhibitor SP600125 to investigate the impact of S1pr2 activation on c-Jun. Our results revealed that S1pr2 activation led to an increase in the phosphorylation level of c-Jun, which was subsequently reduced significantly upon intervention with the JNK inhibitor SP600125 (Figures 6E, F). These findings suggest that S1pr2 modulates the levels of c-Jun phosphorylation via JNK phosphorylation. Furthermore, using siRNA technology, we downregulated c-Jun expression in endothelial cells. Transfection was performed in cells using a scrambled vector and different sequences of c-Jun siRNA. The findings indicated that the RNA expression level of c-Jun was lowest in the c-Jun siRNA-3 group (Figure 6G). Consequently, c-Jun siRNA-3 was chosen as the inhibitory group for further investigation. The results showed that the S1pr2-OE group significantly increased MMP-9 expression while concurrently reducing Occludin and ZO-1 expression. In contrast, the S1pr2-OE + si_c-Jun group exhibited a noticeable reversal of these trends (Figures 6H–K). These results suggest that the activation of S1pr2 exacerbates the disruption of the BBB through the JNK/c-Jun pathway after TBI. Recent studies have confirmed that the biological activity of c-Jun relies on its translocation from the cytoplasm to the nucleus (Wu and Cederbaum, 2008). Two transcriptional cis-acting elements of AP-1 (−31/−24 bp and −467/−460 bp) within the promoter region of the murine MMP-9 gene were identified in previous research (Liao et al., 2020). A chromatin immunoprecipitation (ChIP) assay, using specific primers spanning these two AP-1 sites, showed that SP600126 significantly decreased the binding between p-c-Jun and AP-1 sites after S1pr2 activation *in vitro* (Figure 6L). Furthermore, the results also demonstrated that the binding between p-c-Jun and AP-1 sites increased significantly during TBI. However, such induction was prevented by treatment with JTE-013 (Figure 6M). Collectively, the data indicates that c-Jun, functioning as a transcriptional regulator, exerts a crucial influence on the control of MMP-9 gene expression that underlies the S1pr2-mediated disturbance of the BBB. At last, a diagram of the molecular mechanism elucidated in this study is shown in Figure 7.

## 4 Discussion

Our present investigations delineated the pivotal role of S1pr2 in the disruption of BBB after TBI. We observed an upregulation of S1pr2 expression in endothelial cells post-TBI through RNA-seq, protein and immunofluorescence assays. Notably, pharmacological inhibition of S1pr2 resulted in amelioration of BBB breakdown, oxidative stress, neuronal apoptosis, lesion volume, and neurological deficits after TBI. To elucidate the downstream signaling pathways through which S1pr2 exerts its function, subsequent RNA-seq analyses unveiled the regulatory impact of S1pr2 on MMP-9 activation. It was found that S1pr2 activation enhances MMP-9 expression, exacerbating BBB impairment and neurological functional deterioration via the JNK/c-Jun pathway.

After TBI, the BBB can be compromised through various mechanisms, including physical disruption from the initial mechanical impact, inflammation-induced damage, and enzymatic degradation of BBB components such as MMPs. These factors contribute to increased BBB permeability, permitting harmful substances to infiltrate the brain parenchyma and ultimately exacerbating secondary brain injury (Johnson et al., 2018; Smith et al., 2018; Faden et al., 2021). While the involvement of MMPs in neurovascular injury post-TBI is well-documented, there remains a notable absence of therapies specifically targeting these enzymes. Therefore, there is an imperative for the development of novel vasoprotective agents that bolster the integrity of compromised cerebral blood vessels, thereby enhancing clinical outcomes in TBI. S1pr2 plays an important role in regulating endothelial function (Zhou et al., 2022). The impact of S1pr2 activation on disease progression appears to depend on the specific pathophysiological context. Prior research has elucidated the substantial involvement of S1pr2 in instigating cerebrovascular permeability and inflammation in neurodegenerative disease (Seyedsadr et al., 2019). However, conclusive evidence regarding the pathological significance of S1pr2 in blood-brain barrier disruption following TBI remains uncertain. Unlike neurodegenerative diseases, TBI results in acute mechanical injury that leads to rapid and often severe dysfunction of the BBB. Investigating the molecular mechanisms linked to S1pr2 in pathophysiological contexts of TBI, along with its subsequent molecular mechanism, will facilitate the exploration of novel targeted therapeutic strategies. It found a notable up-regulation of S1pr2 expression through RNA-seq and protein assays post-TBI both *in vivo* and *in vitro*. We identified that TBI triggered the activation of S1pr2, impacting the levels of tight junction proteins, such as ZO-1 and Occludin in endothelial cells. Notably, the inhibition of S1pr2 mitigated secondary damages post-TBI, indicating the pivotal role of S1pr2 in BBB permeability and brain injury after TBI.

In the acute phase following TBI, cerebral MMP-9 is prominently upregulated and exerts a predominant role in mediating cerebral vascular permeability by degrading tight junction proteins such as Occludin and ZO-1 (Sunny et al., 2024). Studies have shown a correlation between serum MMP-9 levels and both neurological deterioration and mortality (DeFazio et al., 2014). Experimental stroke studies have revealed the crucial involvement of MMP-9 in blood-brain barrier permeability (Ji et al., 2023). The suppression of MMP-9 activity, identified in both *in vivo* and *in vitro* conditions, shows a direct link to diminished gelatinase activity in cerebral microvessels, ultimately enhancing the integrity of the BBB and improving neurological function (Chaturvedi and Kaczmarek, 2014). Furthermore, research has highlighted a substantial upsurge in MMP-9 expression levels during TBI, playing a pivotal role in the degradation of extracellular matrix proteins and closely linked to compromised blood-brain barrier function (Sunny et al., 2024). In our research, we observed that the utilization of TNF-α effectively enhances S1pr2 expression, aligning with prior studies (Kim et al., 2015; Zhang et al., 2013). Transcriptome analysis in human brain microvascular endothelial cell lines unveiled a notable shift in MMP-9 expression following treatment with TNF-α compared to TNF-α + JTE-013, suggesting that MMP-9 may serve as a downstream target molecule mediating the biological functions of S1pr2 in the setting of TBI. Individual S1prs exert their diverse cellular functions by interacting with MAPK proteins, thereby initiating a plethora of downstream signaling cascades, including ERK1/2, p38 MAPK, and JNK (Yang et al., 2020; Ye et al., 2021). Moreover, JNK has been demonstrated to link BBB disruption and neuronal apoptosis in various neurological conditions such as stroke and neurodegenerative diseases (Sun et al., 2015; Chiu et al., 2020). Other studies have found that JNK phosphorylates c-Jun to regulate MMP-9 transcriptional activity in the intracerebral hemorrhage (ICH) model (Lu et al., 2022). However, the mechanism by which S1pr2 regulates MMP-9 expression in the pathological environment of TBI remains unclear. Our research identified a pivotal role of JNK phosphorylation in the process of S1pr2-mediated MMP-9 regulation following TBI. Specifically, the activation of S1pr2 induced by TBI led to JNK phosphorylation, with inhibition of S1pr2 resulting in reduced JNK phosphorylation, as evidenced by both animal and cellular studies.

In addition, JNK serves as the principal kinase for c-Jun, which in turn functions as the primary downstream target of JNK (Wang B. et al., 2022). The growing body of evidence emphasizes the crucial role of JNK in phosphorylating c-Jun in various central nervous system conditions (Anfinogenova et al., 2020). Researchers have discovered that the activation of the JNK/c-Jun pathway is associated with neuronal cell death and inflammatory responses, suggesting that this pathway holds potential as a viable therapeutic target for mitigating cerebral injury following a cerebrovascular accident (Yang et al., 2017). In Alzheimer’s disease, increased JNK activity leads to the phosphorylation of c-Jun, thereby intensifying neuronal cell death and contributing to the disease’s progress (Anfinogenova et al., 2020). We demonstrated a significant increase in c-Jun phosphorylation levels in both *in vivo* and *in vitro* experiments following TBI. Importantly, inhibiting JNK phosphorylation significantly decreases c-Jun phosphorylation, highlighting the potential pathological significance of JNK/c-Jun cascade activation in TBI. Our findings also revealed that knockdown of c-Jun inhibited the MMP-9 expression and BBB disruption after S1pr2 activation, suggesting that S1pr2 exerts its disruptive effect on the BBB through the JNK/c-Jun cascade. Notably, c-Jun functions as a subunit of the AP-1 transcription factor and regulates the transcription of different genes associated with inflammation, apoptosis, and proliferation (Zhang J. et al., 2021). In our investigation, a ChIP assay using specific primers spanning these AP-1 sites demonstrated a significant binding between phosphorylated c-Jun and the AP-1a and AP-1b sites both *in vivo* and *in vitro* experiments.

However, our study has some limitations. Firstly, although our animal model of TBI is widely used, it does not fully replicate the complexities of human brain injuries. For instance, the timing and severity of the injury, as well as the specific molecular responses, may differ in clinical settings. Therefore, caution should be exercised when extrapolating our findings to human conditions and further studies should consider utilizing more advanced models such as organoid systems or larger animal models for validation. Secondly, while our findings provide valuable insights into the role of S1pr2 in BBB disruption, clinical validation is crucial. It is imperative to translate our results into clinical studies to assess the relevance of the S1pr2/JNK/c-Jun/MMP9 pathway in human TBI cases. Thirdly, further research is required to elucidate the safety and efficacy of S1pr2 inhibitors in clinical practice, as well as to determine the optimal dosing. This will involve utilizing a broader range of TBI models prior to advancing to clinical trials. Additionally, there is a need for pharmacokinetic and pharmacodynamic studies to comprehensively understand the systemic effects of S1pr2 inhibition and its potential impact on other physiological pathways.

In summary, our data suggest that S1pr2 plays a pivotal role in the disruption of neurovascular integrity following TBI. These findings reveal that the upregulation of S1pr2 enhances the expression of MMP9 in endothelial cells, leading to the disruption of the BBB after TBI through the JNK/c-Jun pathway. Overall, our findings propose that S1pr2 and downstream molecular pathways may represent valuable therapeutic targets for treating TBI.

## Data Availability

The data presented in the study are deposited in the SRA repository, accession numbers PRJNA1186107 and PRJNA1186109.
