# Supplementary material for: Overexpression of endothelial S1pr2 promotes blood–brain barrier disruption via JNK/c-Jun/MMP-9 pathway after traumatic brain injury in both in vivo and in vitro models
Source: Front Pharmacol. 2024 Nov 29;15:1448570. doi: 10.3389/fphar.2024.1448570 (PMC11637860; doi:10.3389/fphar.2024.1448570)
Supplement: Supplementary file 1 [file Table1.DOCX]

**Table S1.** Primer sequences used in RT-qPCR

| Gene | Forward | Reverse |
| --- | --- | --- |
| S1PR1 | ATGGTGTCCACTAGCATCCC | CGATGTTCAACTTGCCTGTGTAG |
| S1PR2 | ACAGCAAGTTCCACTCAGCAA | CTGCACGGGAGTTAAGGACAG |
| S1PR3 | ACTCTCCGGGAACATTACGAT | CCAAGACGATGAAGCTACAGG |
| S1PR4 | TGC GGG TGG CTG AGA GTG | TAG GAT CAG GGC GAA GAC C |
| S1PR5 | CTT AGG ACG CCT GGA AAC C | CCC GCA CCT GAC AGT AAA TC |
| MMP2 | AAGTGGGACAAGAACCAGATC | GATTCGAGAAAACCGCAGTG |
| MMP9 | AGGGGCGTGTCTGGAGATTC | TCCAGGGCACACCAGAGAAC |
| MMP14 | CATCTGTGACGGGAACTTTGA | GGCAGTGTTGATGGACGCA |
| GADPH | CGGAGTCAACGGATTTGGTCGTA | AGCCTTCTCCATGGTGGTGAAGAC |
