# Supplementary material for: Overexpression of endothelial S1pr2 promotes blood–brain barrier disruption via JNK/c-Jun/MMP-9 pathway after traumatic brain injury in both in vivo and in vitro models
Source: Front Pharmacol. 2024 Nov 29;15:1448570. doi: 10.3389/fphar.2024.1448570 (PMC11637860; doi:10.3389/fphar.2024.1448570)
Supplement: Supplementary file 2 [file Table2.DOCX]

**Table S2.** Sequences of siRNA used in cell transfection

| Gene | Sequence (5’ - 3’) | |
| --- | --- | --- |
| c-jun-si-1 | Forward | GGCACAGCUUAAGCAGAAA |
|  | Reverse | UUUCUFCUUAAGCUGUGCC |
| c-jun-si-2 | Forward | GGUGCCUACGGCUACAGUA |
|  | Reverse | UACUGUAGCCGUAGGCACC |
| c-jun-si-3 | Forward | GGAUCAAGGCAGAGAGGAA |
|  | Reverse | UUCCUCUCUGCCUUGAUCC |
