# Supplementary material for: Overexpression of endothelial S1pr2 promotes blood–brain barrier disruption via JNK/c-Jun/MMP-9 pathway after traumatic brain injury in both in vivo and in vitro models
Source: Front Pharmacol. 2024 Nov 29;15:1448570. doi: 10.3389/fphar.2024.1448570 (PMC11637860; doi:10.3389/fphar.2024.1448570)
Supplement: Supplementary file 3 [file Table3.DOCX]

**Table S3.** The primer sequences used in ChIP assays

| Gene | Type | Sequence(5’ to 3’) | Note | Amplicon size (bp) |
| --- | --- | --- | --- | --- |
| AP-1a | F | GTAAACACACACACACACACAC | CHIP | 157 |
|  | R | CCGCAGCTTCTGGCTAAC | CHIP |  |
| AP-1b | F | CCCAAATCCTGCCTCAAAGA | CHIP | 98 |
|  | R | TGTAGGTTCTATCCTCTCATCCC | CHIP |  |
